# Increasing the use of evidence in health policy: practice and views of policy makers and researchers

**DOI:** 10.1186/1743-8462-6-21

**Published:** 2009-08-24

**Authors:** Danielle M Campbell, Sally Redman, Louisa Jorm, Margaret Cooke, Anthony B Zwi, Lucie Rychetnik

**Affiliations:** 1 The Sax Institute, Level 8, Bld 10, 235 Jones St, Ultimo, NSW, Australia; 2 Honorary Associate, Centre for Midwifery and Family Health, University of Technology Sydney, NSW, Australia; 3 School of Public Health and Community Medicine, University of New South Wales, NSW, Australia; 4 Sydney Health Projects Group, University of Sydney, NSW, Australia

## Abstract

**Background:**

Better communication is often suggested as fundamental to increasing the use of research evidence in policy, but little is known about how researchers and policy makers work together or about barriers to exchange. This study explored the views and practice of policy makers and researchers regarding the use of evidence in policy, including: (i) current use of research to inform policy; (ii) dissemination of and access to research findings for policy; (iii) communication and exchange between researchers and policy makers; and (iv) incentives for increasing the use of research in policy.

**Methods:**

Separate but similar interview schedules were developed for policy makers and researchers. Senior policy makers from NSW Health and senior researchers from public health and health service research groups in NSW were invited to participate. Consenting participants were interviewed by an independent research company.

**Results:**

Thirty eight policy makers (79% response rate) and 41 researchers (82% response rate) completed interviews. Policy makers reported rarely using research to inform policy agendas or to evaluate the impact of policy; research was used more commonly to inform policy content. Most researchers reported that their research had informed local policy, mainly by increasing awareness of an issue. Policy makers reported difficulty in accessing useful research syntheses, and only a third of researchers reported developing targeted strategies to inform policy makers of their findings. Both policy makers and researchers wanted more exchange and saw this as important for increasing the use of research evidence in policy; however, both groups reported a high level of involvement by policy makers in research.

**Conclusion:**

Policy makers and researchers recognise the potential of research to contribute to policy and are making significant attempts to integrate research into the policy process. These findings suggest four strategies to assist in increasing the use of research in policy: making research findings more accessible to policy makers; increasing opportunities for interaction between policy makers and researchers; addressing structural barriers such as research receptivity in policy agencies and a lack of incentives for academics to link with policy; and increasing the relevance of research to policy.

## Background

Evidence from research can enhance policy development by identifying new issues for the policy agenda, informing decisions about policy content and direction, or by evaluating the impact of policy [1-5]. Although evidence from research is only one of the many factors considered in policy development, there is an increasing recognition of its potential value. In a recent speech to senior public servants, the Australian Prime Minister identified seven key aspects of policy making, one of which was the use of evidence; he went on to emphasise the importance of evidence-based policy making as part of a robust culture of policy contestability [6].

However, it is evident that many opportunities to use evidence from research in policy are currently missed [7-10], with some authors suggesting that the consideration of evidence by policy makers is 'haphazard' at best [3,11-14]. Grol and Grimshaw conclude, for example, that "*one of the most consistent findings in research of health services is the gap between evidence and practice*" (p. 1225) [15].

A lack of communication, exchange and understanding between researchers and policy makers is often regarded as a major contributor to the failure to consider the relevant evidence. This has been well described by Lomas who noted that "*efforts by researchers and by decision makers seem to proceed largely independently. Both have their own (often misplaced) ideas about the other's environment. Opportunities for ongoing exchange and communication are few. ...It is like two people trying to assemble a jigsaw puzzle, each with half the pieces - but each working in a separate room*" (p. 439) [16].

Relatively little is known about the ways in which researchers and policy makers work together or about barriers to increasing exchange. A systematic review of 24 studies of health policy makers' perceptions indicated that the most important facilitator of research use was personal two-way communication between researchers and policy makers [3]. Research suggests that evidence is more likely to be used to inform policy if it is relevant and accessible [14,17] and there are incentives supporting its consideration [3,14]. However, past studies in this field are mostly surveys of opinions rather than accounts of current use, communication or exchange.

Even less is known about the views of researchers about factors that might increase their participation in policy relevant research and engagement with policy makers. Only one study exploring the views of health researchers was located; it reported that researchers were concerned about the risks posed to an academic career by spending time on engagement with policy agencies [18]. In Australia, tenure and promotion within the higher education system depends on publication in peer-reviewed journals as opposed to broader knowledge transfer activities, and there is a general lack of administrative and monetary support for translation-oriented work.

There are very few studies of engagement between researchers and policy makers in Australia. In 1995, Ross examined the use of economic evaluations by senior policy makers (n = 34) from the New South Wales (NSW) Health Department and the Commonwealth Health Department and found that only 38% of the policy makers had ever used economic evaluations to inform policy development [19]. A survey of population health staff in an Area Health Service in NSW found that most felt that there was a lack of evidence on which to base policy and that an evidence based approach would improve practice [20]; relatively few reported using a web-based portal, the Clinical Information Access Program, to access evidence about population health interventions [21].

A recent study of reports from completed projects funded through National Health and Medical Research Council (NHMRC) grants found that only 14% of principal investigators felt their research had influenced public health practice, and only nine percent believed their work had made any impact on health policy [22]. An earlier survey of NHMRC grant recipients asked about the potential value of different approaches to enhancing research dissemination [23]. The most highly rated suggestions included encouraging greater demand for research findings - and better skills at judging 'good' research - among policy makers, encouraging policy makers to become involved in research, better infrastructure support for public health research, and more research funded by health organisations.

This study aimed to explore the views and current practice of both policy makers and researchers about the use of evidence in policy. Specifically, the study aimed to:

i. Describe the extent to which policy makers and researchers believed that research is currently used to inform policy

ii. Investigate current practice in relation to the dissemination of and access to research findings for policy

iii. Explore the extent of communication and exchange between researchers and policy makers and

iv. Examine incentives for increasing the use of research in policy

## Methods

### Setting

The Sax Institute was established in 2002 with core funding from the NSW Department of Health. The Institute is a unique organisation in Australia that aims to build excellent policy- and practice-focused health research and increase the impact of this research on health policy, programs and services. The Institute is an independent, not-for-profit organisation structured as a coalition of member organisations. Membership is open to Universities, Schools and research groups with programs in public health and health services research. At the time of the study the Institute's members included 34 research groups (six Universities and 28 Schools/research centres) of national and international standing, representing most of the Universities and research groups that undertake public health and health services research in NSW. A full list of members and details about membership are available on the Sax Institute's website .

### Procedure

The study was designed as a quality assurance exercise to assess, and inform the further development of, the Sax Institute's programs for improving links between research and policy. The study complied with the definition of a quality assurance activity as set out in the relevant National Health and Medical Research Council guidelines [24]. Specifically, participants were being interviewed in their professional capacity to describe their current practice; the interviews were undertaken with the informed consent of participants; participants were unlikely to suffer burden or harm; and no personal details or identifying information were collected. Accordingly, the approval of a human research ethics committee was not sought.

Separate interview schedules were prepared for researchers and policy makers and samples identified as described below. The procedure for administering both sets of interviews was the same. Structured telephone interviews were conducted by trained interviewers from an independent research company. Potential participants were initially contacted by telephone and asked to identify a suitable interview time; a minimum of six follow-up call attempts were made to establish contact. Participants were informed that their responses would be fully de-identified. Interviews took an average of 30 minutes to complete. The interviews were not recorded, but responses to open-ended questions were handwritten verbatim and subsequently coded using thematic analysis to identify common categories.

### Interviews with policy makers

#### Participants

All members of the NSW Department of Health Policy Development Committee and all directors of Health Service Development and of Population Health from each of the Area Health Services were sent a letter of invitation from the NSW Chief Health Officer (n = 54).

#### Interview schedule

The interview schedule included both closed and open-ended questions and asked about respondents' involvement in policy development, access to and use of research findings, and involvement in research activities and networks. Participants were asked to think broadly about policy and to include in their answers policy in the form of small-scale local plans or operational issues through to large-scale programs or system-wide directions, and relating to a variety of issues including resource allocation, service patterns, or the delivery of health care or public health programs. In responding to questions about research, participants were asked to include any kind of formal or systematic process of collecting and analysing data, including stand-alone studies, studies that form part of a broad thematic program of research, and research reviews.

### Interviews with researchers

#### Participants

Sixty senior population health and health services researchers were invited to participate through a letter sent from the Chief Executive Officer of the Sax Institute. Invitees included nominated representatives from the Institute's member Universities (n = 6), nominated members from the Institute's member Schools and research centres (n = 28), and one additional nominee from each member School and research centre currently employing two or more senior researchers (n = 26).

#### Interview schedules

The structured interview schedule included both closed and open-ended questions and asked about respondents' involvement in policy development, dissemination of their research and its impact on policy, and degree of involvement of policy makers in their research. The descriptions of the terms 'policy' and 'research' were the same as those provided in the interviews with policy makers.

## Results

### Sample

#### Policy makers

Of the 54 people approached, six people were on extended leave or had transferred from their area. Of the remaining 48 potential participants, 10 did not respond to the letter of invitation. The final sample consisted of 38 interviewees (79% response rate).

The 38 policy makers interviewed were employed at senior levels of the NSW Department of Health (n = 14) and the Area Health Services (n = 24). Over half (58%) had worked in their current position for more than two years. All participants had been involved in policy development in the last 12 months, with 71% having developed more than five policies and 84% having approved policies developed by other staff. Respondents were involved in developing a range of policies relating to population health (eg state wide immunisation strategy, Area-level public health plan), health service provision (eg cancer services plan), governance and administration (eg patient information privacy), and clinical care (eg collection of urine samples for testing).

#### Researchers

Of the 60 researchers approached, six were unavailable during the study period and four no longer held a substantive research position. Of the remaining 50 potential participants, five opted out of the study and four could not be contacted. Forty-one researchers completed an interview (82% response rate). The 41 researchers interviewed were drawn from 29 of the Sax Institute's member organisations across NSW. All but one respondent (98%) had worked in an academic research environment for eight or more years (range 3 to 40 years, mean 19 years). Interviewees identified their primary research areas as public health (56%), health services (51%), clinical and medical sciences (27%), and equity (12%).

### Is research currently used to inform policy?

#### Policy makers

Respondents were asked to indicate how much of a need there is to increase the use of research in policy making using a five point scale. Sixty three percent of respondents felt that there was a high need to increase the use of research (rated 4 or 5) and a further 24% believed that there was a medium need (rated 3).

**Figure 1:**
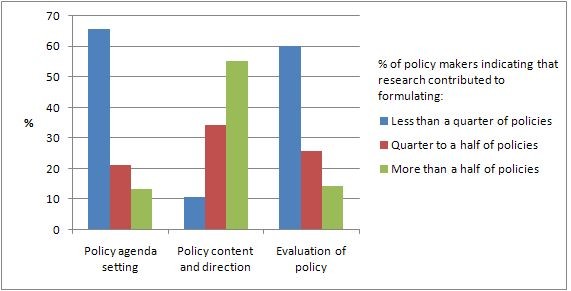
**Policy makers' views about the impact of research on: (a) policy agenda setting; (b) policy content/direction; and (c) evaluation**.

Figure 1 shows respondents' reports of their use of research in policy in the past 12 months in terms of getting issues onto the policy agenda, informing policy content and direction, and evaluating the implementation or the impact of a policy.

Respondents were asked to estimate, for those policies that they had developed or approved, whether research was used to inform: (a) none of these policies; (b) less than a quarter; (c) between a quarter and a half; (d) between half and three quarters; or (e) more than three quarters. The majority of respondents used research only infrequently to inform policy agendas. Sixty six percent of respondents used research in agenda setting on less than a quarter of occasions in the previous year; this included two participants who had never used research to inform policy agendas. Most participants also used research infrequently to evaluate the implementation or impact of policies: 60% had used research to evaluate policies on less than a quarter of occasions in the previous year. This included three participants who had never used research to evaluate policies.

Use of research to inform policy content or direction was more common. All respondents had used research to inform policy content at some time in the previous year, and only 11% reported using research on less than a quarter of policies. Nevertheless, only a minority of participants (29%) used research to inform content on more than three-quarters of policies.

#### Researchers

Eighty five percent of respondents perceived a high need to increase research use by policy makers (rated 4 or 5) and the remainder believed that there was a moderate need (rated 3).

Table 1 shows researchers' reports of the impact of their research on policy. Two thirds of the respondents reported that findings from their research had been used to inform health policy or practice within NSW, and 69% agreed that a research review they had undertaken in the previous two years had impacted on policy in some way. Thirty nine percent could identify findings from their research that they believed should have been used to inform local policy or practice, but which had not been used. When asked to describe *how *their research had influenced health policy or practice, respondents most often felt that their research or reviews had increased policy makers' awareness of an issue.

**Table 1:** Researchers' views about the impact of research on policy or practice and reported dissemination

	**Research findings** *(n = 41)*		**Research review** *(n = 29)*^1^	
**IMPACT OF RESEARCH ON POLICY**	**n**	**%**	**n**	**%**

Own research or reviews have been used to inform policy or practice	27	66	20	69
Had findings that were not used by policy but were potentially important	16	39	NA	NA
Own research increased policy makers' awareness of the issue	24	59	19	66
Own research helped to get the issue on the policy agenda	20	49	13	45
Own research assisted policy makers in formulating the policy issue	19	46	14	48
Own research helped policy makers to identify policy alternatives	17	41	11	38
Own research helped policy makers to choose the preferred policy option	20	49	9	31
Own research used to justify the final policy	20	49	12	41

**DISSEMINATION**^2^				

Published in peer review journal	36	88	9	31
Published in report for funding agency	27	66	16	55
Published in report for stakeholders	22	54	10	34
Presented at conference	37	90	10	34
Presented to policy makers	21	51	13	45
Presented to practitioners	26	63	8	28

^1^Researchers who had contributed to a review in the previous two years

^2^Most recent research paper or review

### Are research findings accessible and useful?

#### Policy makers

Respondents were asked whether the health research undertaken by researchers in NSW was relevant to policy and program development. Over one third of respondents (39%) felt that local research was relevant, but most of these (87%) believed that the research was not presented in a useful way. In total, only 5% of the interviewees felt that local research was both relevant and presented in a useful way.

**Figure 2:**
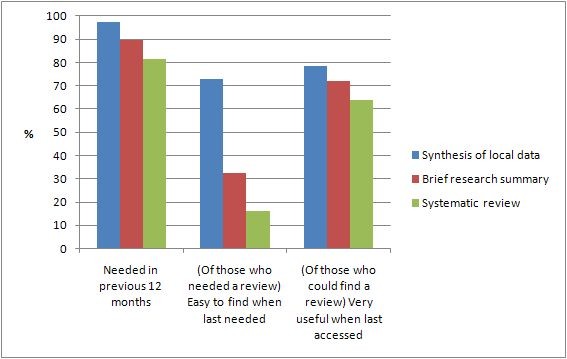
**Policy makers' need for and use of research syntheses in the previous 12 months**.

Figure 2 shows participants' reports about their use of three different types of research syntheses over the past 12 months. It is evident that policy makers needed all three types of synthesis, but had difficulty finding brief research summaries and systematic reviews when they were needed. Policy makers who accessed research syntheses reported that these were almost always of some use in terms of decision making. Summaries of local data were rated as highly useful by 78% of these respondents, with 72% rating brief research summaries and 64% rating systematic reviews as highly useful respectively.

#### Researchers

Respondents were asked how often they had used various strategies to communicate their research findings in the previous two years. Twenty three (56%) reported that they often identified the policy or practice implications of their research findings, but only 14 (34%) regularly developed explicit policy recommendations or summaries from their research for policy makers. Sixteen (39%) respondents had frequently developed targeted strategies for communicating their research to non-academic audiences, and 18 (44%) often wrote reports or papers about their research for non-academics.

Table 1 shows respondents' reports of dissemination in relation to the most recent research study or review in which they had been involved. Although the most common dissemination strategies were conference papers and peer review papers, half reported a presentation of findings to policy makers.

### How much communication and exchange is there between researchers and policy makers?

#### Policy makers

Most policy makers (n = 28, 74%) had wanted to contact a researcher during the past 12 months to sound out an issue. Of those who had wanted to discuss ideas with a researcher in the previous year, 57% (42% of the total sample) were easily able to contact a relevant researcher when needed.

Table 2 shows policy makers' communication and exchange with researchers in the past 12 months. Most respondents had attended forums to hear research findings and about half of the respondents had actively invited researchers to participate in the policy process by providing a research perspective or joining a policy development committee.

**Table 2:** Exchange between researchers and policy makers

**POLICY MAKERS' REPORTED EXCHANGE WITH RESEARCHERS**		
**Links with researchers in the previous 12 months (and reports that these links were useful^1^) ***(n = 38)*	**n**	**%**

Attended forums with researchers and policy makers to hear about research findings	33 (12)	87 (32)
Invited researchers to give a research perspective in an area of policy development	22 (15)	58 (39)
Invited researchers to be an active member of a policy development committee	18 (14)	47 (37)
Regularly used research contacts as a sounding board for policy work	28 (20)	74 (53)
Contracted a research group or individual to conduct a research review or study	20 (15)	53 (39)

**Involvement in research in the previous 12 months ***(n = 38)*	**n**	**%**

Acted in an advisory capacity to a research team	26	68
Contributed to the development of research questions	28	74
Collaborated on a successful competitive research grant	7	18
Active member of a research team	18	47
Collaborated in analysis and writing up of findings	16	42
Authored or co-authored a research publication	12	32
Assisted in dissemination	25	66

**RESEARCHERS' REPORTED EXCHANGE WITH POLICY MAKERS**		

**Involvement in policy activities in the previous two years ***(n = 41)*	**n**	**%**

Presented research findings at a forum where NSW policy makers likely to have been present	32	78
Presented research findings at a forum organised specifically for NSW policy makers	21	51
Actively participated on a health policy development committee	23	56
Used as a 'sounding board' by a policy maker to provide a research perspective	27	66
Funded by NSW policy agency to conduct a review of research	12	29
Funded by NSW policy agency to conduct a research project	21	51

**Involvement of policy makers in research in the previous two years (and reports that this involvement was useful^1^) ***(n = 41)*	**n**	**%**

Acted in an advisory capacity to a research team	28 (16)	68 (39)
Discussed ideas for research questions	29 (16)	71 (39)
Collaborated on a competitive research grant	25 (13)	61 (32)
Active member of a research team	20 (8)	49 (20)
Collaborated on analysis or writing up of results	18 (5)	44 (12)
Co-authored research publication	17 (7)	41 (17)
Assisted in disseminating results	18 (15)	44 (37)

^1^Rated 4 or 5 on a 5-point scale

More than two-thirds of the policy makers had acted in an advisory role in research, participated in the development of research questions, or assisted with the dissemination of research results. Half of the interviewees reported active participation in a research team. However, fewer had been involved in the sorts of activities that are likely to facilitate communication and application of research, such as participating in the analysis, writing up and publication of the research results. Eighteen percent of the sample had collaborated on a successful competitive research grant.

#### Researchers

Table 2 also shows researchers' reports about communication with policy makers in the last two years. Almost all had presented their research findings at a conference or forum where state- or Area-level policy makers were likely to have been present, but only half had presented their research findings to a forum specifically for policy makers. Two thirds had been approached informally to provide a research perspective on a policy issue, half had been funded by a local policy agency to conduct research, and a third had been funded to undertake a research review.

Eighty percent of interviewees had wanted to involve a policy maker in their research at some time in the previous two years. Of these, 58% (46% of the total) were easily able to find a policy maker to contribute when needed, but 27% found it difficult to contact a policy maker and 15% could not find an appropriate person. For those interviewees who did find a policy maker to contribute to their research, this was almost always (79%) based on an existing relationship.

Table 2 summarises the ways in which respondents had involved policy makers in their research in the previous two years. Policy makers were most often approached by researchers to sound out ideas for research questions or act in an advisory capacity to a research team, such as through a steering committee. Respondents felt that these were the most useful roles for policy makers in terms of influencing the direction, implementation, interpretation or dissemination of their research.

### What could be done to increase the use of research in policy?

#### Policy makers

In response to an open question, the most common reasons for not using research in policy were: the absence of appropriate and/or relevant research (29%); a lack of skills or capacity to access or acquire relevant research (24%); the need to consider local agendas and other policy drivers (24%); and time pressures (21%). The most frequently nominated strategy for improving the use of research in respondents' organisations was improved access to research and researchers (32%). Participants' suggestions included: "*building bridging systems between researchers and policy makers*" and "*standing arrangement with key research groups and key research people who can readily assist in policy making*".

Fifty five percent of the respondents were not aware of a NSW Health guideline that required evidence to be checked during policy development. Forty two percent of the sample perceived that NSW Health placed a high value (rated 4 or 5 on a five point scale) on policy being supported by research.

#### Researchers

Of the 27 researchers who reported that their research had been used in policy, the most commonly cited facilitators identified in response to an open question were: existing relationships and networks with policy makers (33%); the quality and credibility of the research (33%); a receptive policy environment - the 'right research at the right time' (33%); and research that was designed specifically to address policy priorities (19%). The 16 researchers who felt that their research should have been but was not used to inform policy reported that the use of their research was impeded by: research findings that were politically sensitive or inconsistent with policy directions (38%); the importance of other policy drivers, such as politics or media (31%); and practical constraints to the implementation of findings, such as financial implications (25%). Respondents' suggestions for improving the impact of their research on policy and practice included: encouraging a better understanding of the importance of research among policy makers and politicians (31%); more opportunities for dialogue and interaction with policy makers (25%); and more research and funding (19%).

Respondents indicated that none of the policy making, research funding or academic sectors provided significant incentives to increase research uptake (Table 3). Thirty eight percent of respondents felt that the NSW Department of Health placed a high value (rated 4 or 5 on a five point scale) on policy being supported by research. Most respondents believed that funding bodies did not value academics' achievements in research transfer when evaluating their track record, and that Universities did not value these achievements when considering promotion.

**Table 3:** Researchers' views about incentives and motivation to improve research uptake

	**None/little**^1^		**Medium**^2^		**High**^3^	
	**n**	**%**	**n**	**%**	**n**	**%**

Perceived value placed by NSW Health on policy being supported by research (*n *= 39)	15	38	9	23	15	38
Perceived value placed by funding bodies on research transfer activities (*n *= 40)	26	65	11	28	3	8
Perceived value placed by universities on research transfer activities (*n *= 41)	31	76	6	15	4	10

^1^Rated 1 or 2 on a 5-point scale

^2^Rated 3 on a 5-point scale

^3^Rated 4 or 5 on a 5-point scale

## Discussion

This paper reports findings from interviews with both senior policy makers and researchers in NSW. While the samples were small, this reflects the size of the relevant research and policy communities in NSW, and the response rates were good.

Policy makers and researchers recognise the potential of research to contribute to policy and are making significant attempts to integrate research into the policy process. Most policy makers reported having needed data and reviews in the past 12 months, having commissioned research or reviews during this period, and having used evidence to contribute to the content of policy. The rates of use of evidence by policy makers appear to be somewhat higher than those reported in previous Australian surveys (eg [19]). Similarly, over the past 12 months, most researchers felt that their work had had a policy impact; in particular, they felt that their research had contributed to raising awareness of issues and to setting policy agendas.

However, policy makers and researchers agreed that much more could be done to increase the use of research in policy. Reports of current practice indicated that only around half of the researchers thought their research had been used to get issues on the policy agenda or select preferred policy options in the past two years. Although policy makers drew on research findings to contribute to the content of policy, it was not often used to set agendas or to evaluate policy.

This paper identifies four potential strategies for increasing the use of research in policy.

First, making research findings more accessible is likely to be helpful. Policy makers reported that they often found it difficult to access brief summaries and systematic reviews. Many respondents also indicated that research conducted in NSW was often not presented in a useful way to inform policy and program issues. Similar results have been reported by others [19,25,26]. The difficulty in accessing reviews was reported despite the fact that NSW Health employees have access to research reviews on the web through the Clinical Information Access Project (CIAP), encompassing a wide range of research databases such as Medline and Cochrane. In contrast, participants reported that they could easily access local data and that these data were very useful. Decision makers at NSW Health have access to data resources including the Health Outcomes Information Statistical Toolkit (HOIST) which houses a wide range of relevant population-based data collections (eg census data and morbidity and mortality data). This system allows manipulation, analysis and reporting of data at the state and regional level. There appears to be a high level of awareness and use of this system and other sources of local health data within the Area Health Services and Department of Health.

Researchers reported a high level of effort in disseminating their research to policy makers. Thirty nine percent of respondents had regularly developed targeted strategies for communicating their findings to non-academic audiences. While peer review papers and conference papers remain the standard methods of dissemination, there is certainly evidence of a second tier dissemination strategy aimed at policy makers, primarily through research reports and presentations.

However, despite these efforts by researchers, policy makers still found it difficult to access research findings. It seems likely that new approaches are required that more closely target the specific needs of policy users [25]. There is evidence that decision makers appreciate very brief summaries of research findings, preferably with a more detailed structured abstract and along with clear statements of implications for practice [27,28]. The Canadian Health Service Research Foundation's (CHRSF) 1:3:25 format (one page of main messages, a three-page executive summary, and a 25-page report), for example, is widely recommended [29].

Second, increasing the opportunities for interaction and exchange between policy makers and researchers is key to promoting the use of research evidence in policy. This was identified by both policy makers and researchers in our samples, consistent with the findings of two systematic reviews [3,30]. Our respondents reported a moderate current level of interaction, indicating that partnership and collaboration is feasible. For example, around half of the researchers reported being involved in policy development committees, being used as a sounding board or being funded by government for policy-relevant research. Likewise, around half of the policy makers indicated that they were involved in some research activities. This was in broad agreement with researchers' views about their involvement of policy makers.

Opportunities for researchers and policy makers to meet informally and mechanisms to help policy makers and researchers to identify individuals relevant to their work are likely to be important in promoting exchange. Policy makers in our sample reported that they often wanted to seek advice from researchers, but sometimes could not find the expertise that they needed, and that they tended to use existing contacts. Researchers perceived that input from policy makers into their research would be of value but were often not sure how best to identify appropriate individuals.

A greater intensity of interaction and exchange is achieved by actively involving policy makers in conceptualising, designing, and implementing research [31]. Experiences with 'research partnership' models in the UK suggest that the involvement of decision makers both focuses the research on users' needs and encourages a better understanding on the part of researchers of the decision making environment [32]. In Australia, funding schemes such as Australian Research Council linkage grants and the National Health and Medical Research Council's new Partnership Awards require the formal involvement of an industry partner, and are important mechanisms for encouraging collaboration between researchers and policy makers. Internationally, the CHRSF's Research, Exchange and Impact for System Support program [33] and United Kingdom's National Institute for Health Research Service Delivery and Organisation Programme [34] might provide models for the future in Australia.

Third, there are clearly some structural barriers to increasing the use of research in policy that could be addressed. Both policy makers and researchers felt that enhancing policy makers' understanding of research is important; likewise, the need to improve research infrastructure and funding was regarded as important in generating relevant evidence. Policy makers felt that organisational reinforcement for evidence-informed policy could be improved. Although researchers agreed that there was a high need to increase the use of research by policy makers, more than one-third of the respondents in the current sample did not regard these activities as being a high personal priority. This is in part the result of a perception among researchers that their efforts to impact on policy were not valued by Universities or by funding agencies. This view is probably well founded; for example, in Canada, Phaneuf et al. [35] surveyed academic promotion committee members and found that they regarded peer review publications as substantially more important in their decision making than work with policy. Although it was not directly examined in this study, it is evident that concerns about intellectual property, independence and the right to publish are also structural barriers to a greater engagement of researchers with policy agencies [36].

There seems little doubt that it will be necessary to address these structural barriers to increase the use of evidence in policy. In terms of increasing the receptivity of policy makers to research, the two main approaches that have been described are the use of tools to assess organisational capacity to acquire and apply research evidence (for example a self-assessment tool developed by the CHRSF [37]) and continuing education programs (for example the CHSRF-sponsored Executive Training for Research Application (EXTRA) program [38]). A relevant Australian example is the Australasian Cochrane Centre (ACC) Policy Liaison Initiative, through which the ACC provides support and training to an Evidence-Based Policy Network within the Australian Government Department of Health and Ageing [39].

With regard to incentives for researchers to engage in research transfer activities, there is a need to develop a measure of the impact of research on policy. A reliable measurement tool would enable these activities to be included in consideration of applications for promotion or in assessment of research track record for funding applications. For example, the Netherlands Council for Medical Sciences has developed a methodology and indicators for evaluating the societal impact of applied health research [40]; this is considerably more sophisticated than the generic measures for research impact proposed as part of the (now abandoned) Australian Research Quality Framework.

Finally, there was a view among policy makers that there is a lack of relevant research that could inform policy. Almost half the sample believed that the health research being conducted in NSW was not relevant, or had variable relevance, to health policy. To increase the relevance of research, policy makers need be able to clearly identify and communicate gaps in knowledge and policy priorities for research to researchers. A greater understanding of the policy context by researchers could increase relevance by focusing the research on more useful questions, collecting information critical for policy decisions (for example on costs) and improving the description of the research results and their implications. Research partnerships may improve the relevance of research and therefore its translation to policy [41].

The development of a national system for health data linkage through the National Collaborative Research Infrastructure Strategy (NCRIS) Population Health Research Network presents particular opportunities for new policy-relevant health research in Australia. Linked person-based data for entire populations provides powerful information about the outcomes of health systems, and how these are shaped both by environmental factors, patient factors and service configuration. However, to provide the information health policy makers need, this enhanced capacity to describe and monitor system outcomes must be accompanied by new multidisciplinary research to develop health service interventions and test these in real-life service settings.

We intend to repeat our policy maker and researcher interviews in 2010. The 2010 sample of policy makers is likely to be almost entirely new, given the rapidity of change within the policy environment. Nonetheless, we would hope to demonstrate increased use of research evidence in health policy in NSW, as a result of the activities of the Sax Institute and initiatives such as the NHMRC Partnerships Program and the NCRIS Population Health Research Network, and reflecting a general, worldwide interest in promoting the efficient transfer of research evidence into policy.

## Competing interests

The authors declare that they have no competing interests.

## Authors' contributions

DC oversaw the design and conduct of the researcher interviews, undertook data analysis, and drafted the manuscript. SR contributed to the conception and design of the study, and to the development, drafting and editing of the manuscript. LJ and AZB contributed to the conception and design of the study, and helped to draft the manuscript. MC oversaw the design and conduct of the policy maker interviews, undertook data analysis, and helped to draft the manuscript. LR contributed to the design of the study and helped to draft the manuscript. All authors read and approved the final manuscript.
